# Novel DNA–Dye Hybrids Sniff out Odors

**DOI:** 10.1371/journal.pbio.0060020

**Published:** 2008-01-22

**Authors:** Richard Robinson

Combinatorial diversity is the central feature of DNA. Because each nucleotide position within the molecule can be occupied by any one of four bases, there are 4^2^ possible dinucleotides (“2mers”), 4^3^ possible trinucleotides (“3mers”), and so on; by the time we reach 21 nucleotides, there are over a trillion possible “21mers,” each with a unique sequence. But the order of bases is not the only thing that distinguishes two DNA molecules. Because bases differ chemically, each unique sequence also differs in its exact shape and in the distribution of charges on its surface. Those trillion 21mers represent a trillion different chemical environments.

While sequence diversity accounts for the diversity of living things, the chemical diversity of DNA has other potential applications. In a new study, Joel White, John Kauer, and colleagues show that a fluorescent dye, linked to a variety of short DNA molecules and dried onto a solid surface, will respond differently to different odor molecules, depending on the exact DNA sequence to which it is attached. Their results show that such DNA–dye hybrids have the potential to act as sensors in an “artificial nose,” detecting and distinguishing among a range of odors.

The authors initially used double-stranded DNA, which was linked to a dye that inserts itself between successive rungs on the DNA ladder and dried onto a solid plastic surface. While the dye preferentially responded to one test odor over others, no sequence specificity was observed. Therefore, they tested single-stranded DNA oligomers, 20 to 24 bases long, which was linked to a different dye and again dried onto plastic. They found that one of 30 test sequences increased its fluorescent glow when exposed to either methanol or propionic acid, but not to other odors, including water and triethylamine.

The first two dyes they tested attached randomly to DNA, making it potentially difficult to study the sensing mechanism. To avoid this, they switched to a dye that was covalently attached to one end of the DNA. They also developed a new sensor screening method that allowed an odor to waft over a large array of different oligomers all on the same substrate; which allowed changes in fluorescence to be detected by standard microarray scanners.

After confirming a high degree of replicability among tests of a single sequence with an odor test set, the authors tested 29 different sequences against the same set of odors. Among these sequences, they found ten groups that responded differently to the odor set. That a relatively large proportion of the initial set of sequences showed different responses suggests that the number of different sensors among larger libraries of small DNA molecules is likely to be enormous. 2,4-dinitrotoluene, found in the vapor signature of TNT from landmines, could be detected down to six parts per billion, indicating the potential for practical and life-saving applications of this technology.

One salient feature of this work is that DNA's utility for this application is entirely independent of the encoding role it plays in a cell—these sequences are never translated into RNA or used to make protein. In theory, some other heteropolymer could create a similar variety of chemical environments to alter the dye's response to odor. But the tools for synthesizing and manipulating DNA are more advanced than those for any other polymer (except perhaps RNA). This study not only highlights DNA's potential as the scaffolding for a novel and powerful odor detection system, but it also highlights its potential to play other novel roles, well outside of its familiar one as the basis of life.

**Figure:**
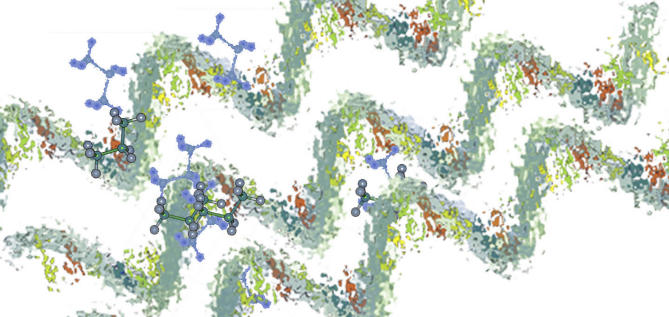
DNA's combinatorial nature and the finding that solid-state DNA responds to vaporized odors reveals a novel function for this intensely studied molecule: its capacity to produce a vast repertoire of odor sensors for an artificial nose system that can detect many different compounds. (Painting credit: Dr. MJ Morse, Manager Science and Technology, Museum of Science, Boston, Massachusetts)

